# Clinical and Prognostic Value of Exaggerated Blood Pressure Response to Exercise

**DOI:** 10.31083/j.rcm2403064

**Published:** 2023-02-22

**Authors:** Cesare Cuspidi, Andrea Faggiano, Elisa Gherbesi, Carla Sala, Guido Grassi, Marijana Tadic

**Affiliations:** ^1^Department of Medicine and Surgery, University of Milano-Bicocca, 20126 Milano, Italy; ^2^Department of Clinical Sciences and Community Health, University of Milano, 20122 Milano, Italy; ^3^Department of Cardio-Thoracic-Vascular Diseases, Foundation IRCCS Ca' Granda Ospedale Maggiore Policlinico, 20122 Milano, Italy; ^4^Department of Cardiology, University Hospital “Dr. Dragisa Misovic-Dedinje'', 11000 Belgrade, Serbia

**Keywords:** exaggerated blood pressure response to exercise, hypertension, target organ damage, cardiovascular disease

## Abstract

The hypertensive response to exercise testing, defined as exaggerated blood pressure response (EBPR), has been documented to be independently associated with unhealthy conditions, carrying an increased risk of future hypertension, cardiovascular (CV) morbidity and mortality. In treated hypertensives, EBPR is a marker of uncontrolled hypertension, a condition previously undetected by office blood pressure (BP) measurements at rest; EBPR may also detect masked hypertension, a phenotype characterized by normal BP values in the medical environment but elevated home or ambulatory BP monitoring (ABPM). The aim of the present review is to provide a comprehensive and up-dated information on the clinical importance of EBPR targeting the following issues: (I) definition and prevalence; (II) underlying mechanisms; (III) clinical correlates and association with subclinical organ damage; (IV) predictive value; (V) clinical decision making.

## 1. Introduction

In both normotensive and hypertensive individuals, physical exercise, either dynamic or isometric, carried out in the clinical setting for cardiovascular (CV) diagnostics, is associated to significant blood pressure (BP) variations, in particular sharp increments in systolic BP (SBP) and variable changes in diastolic BP (DBP), that may either decrease, increase or remain unchanged [1, 2]. In physiological conditions, BP changes during exercise are the result of the rise in cardiac output in response to the increased oxygen demand from working muscles via activation of the adrenergic tone [3, 4]. The electrocardiography (ECG)-monitored stress test for assessing cardiopulmonary fitness is routinely performed by means of dynamic exercise with a progressive workload. In response to the rapid increase in physical activity, stroke volume and heart rate are boosted by increased sympathetic activity and a vasodilation occurs at the level of arterioles supplying the exercising muscles, thus leading to a decrease in systemic vascular resistances [5].

Stress ECG testing with measurement of BP at incremental stages of exercise intensity is a validated non-invasive diagnostic tool carried out worldwide in cardiology practice [6, 7]. Measuring BP response to physical exercise during stress ECG testing represents a key procedure, as abnormal responses in terms of BP increases or decreases provide relevant information in addition to conventional ECG and clinical diagnostic criteria [8, 9]. In addition to dynamic exercise, other stimuli such as mental stress, cold stress and handgrip represent an alternative method for evaluating exaggerated pressure reactivity [10]. Exaggerated BP response (EBPR) to these stimuli has been also associated with poor prognosis; our review, however, will focus on the abnormal pressure reactivity to exercise ECG-testing [11].

Consistent evidence indicates that hypertensive response to exercise testing is independently associated with several CV risk factors and, more importantly, with an increased likelihood of CV morbidity and mortality [12, 13, 14, 15]. The strength of this association is more evident when EBPR occurs in the early stages of test or during submaximal exercise [16, 17]. Of note, EBPR in treated hypertensives may be seen as a marker of uncontrolled hypertension previously undetected by office BP measurements at rest [18]. On the other hand, EBPR in normotensive individuals may be a marker of masked hypertension and predictor of future hypertension [19, 20]. This review aimed to summarise available evidence about the clinical relevance of EBPR will be focused on the following issues: (I) definition and prevalence; (II) mechanisms; (III) clinical correlates and association with subclinical organ damage; (IV) prognostic value; (V) clinical management.

## 2. Definition and Prevalence

Several definitions of EBPR during treadmill and bicycle exercise testing have been reported in the literature; this may be relate to the lack of consensus about the ‘threshold’ value of exercise BP defining this phenotype. This uncertainty is also reflected by the different recommendations issued by the Cardiology and Sports Medicine guidelines. The American Heart Association (AHA) guidelines define EBPR as systolic peak BP >210 mmHg in men and >190 mmHg in women, and/or diastolic peak >90 mmHg in both sexes; the corresponding thresholds recommended by the European Society of Cardiology (ESC) guidelines are systolic peak >220 mmHg in men, >200 mmHg in women and/or diastolic peak >85 mmHg in men, >80 mmHg in women [21, 22]. At difference, the American College Sports Medicine (ACSM) guidelines suggest a non-sex specific BP threshold (i.e., systolic peak BP >225 mmHg and/or diastolic peak BP >90 mmHg) [23] (Table 1). Different definitions of EBPR are based on SBPs values exceeding either the 90th or 95th percentile of the study population. Recently, age- and sex-specific peak SBP 90th percentiles have been used to assess the risk of CV morbidity and mortality associated with EBPR [24].

**Table 1.** **Blood pressure diagnostic cut-offs recommended by guidelines to define exaggerated blood pressure response to exercise**.

Guidelines	Blood pressure cut-offs	
	Men	Women
American Heart Association	210/90 mmHg	190/90 mmHg
European Society of Cardiology	220/85 mmHg	200/80 mmHg
American College Sports Medicine	225/90 mmHg	

A meta-analysis of 12 studies revealed that EBPR may be defined over a wide interval of SBP values ranging from 180 to 275 mmHg [25]. A further source of heterogeneity of EBPR diagnostic criteria is the intensity of physical exercise. Differences in protocol, type of exercise (i.e., bicycle, treadmill), age, gender, degree of physical activity of the individuals examined (i.e., sedentary, trained, athletes) and comorbidities are major determinants of variability among studies. A consequence of the lack of consensus about EBPR definition is the difficulty in comparing and interpreting data provided by the studies. It is worth noting that EBPR may occur in both normotensive individuals with no known history of hypertension and in treated hypertensive patients. Thus, the variable prevalence of EBPR reported in current literature may be related to numerous factors including threshold values used to define this phenotype, demographic (i.e., age, sex, ethnicity) and clinical characteristics of the populations studied. In the Southall and Brent Revisited (SABRE) study, including 659 older adults, EBPR (i.e., SBP ≥210 mmHg in men and >190 mmHg in women and/or DBP ≥110 mmHg in both sexes) was found in 31% of normotensive and 23% of hypertensive participants with controlled resting BP [26]. The EXERTION study (Exercise Stress Test Collaboration) based on the results of clinical stress testing recorded from multiple hospitals in Australia and including 13.268 subjects (aged 53 ± 13 years, 9% with type 2 diabetes) who completed the Bruce treadmill protocol, showed that EBPR overall prevalence at stage 3 in participants with ‘normal’ pre-exercise BP (<140/90 mmHg) was 6.4% [13]. In a large cohort of 1167 competitive athletes of any age without known arterial hypertension, EBPR prevalence rates varied from 6.8 to 19.6% according to ACSM and ESC guidelines, respectively [27]. It is therefore evident that EBPR during physical exercise may affect a significant fraction of individuals with normal BP at rest, EBPR prevalence ranging from 5 to 30%.

## 3. Mechanisms

**Fig. 1.**
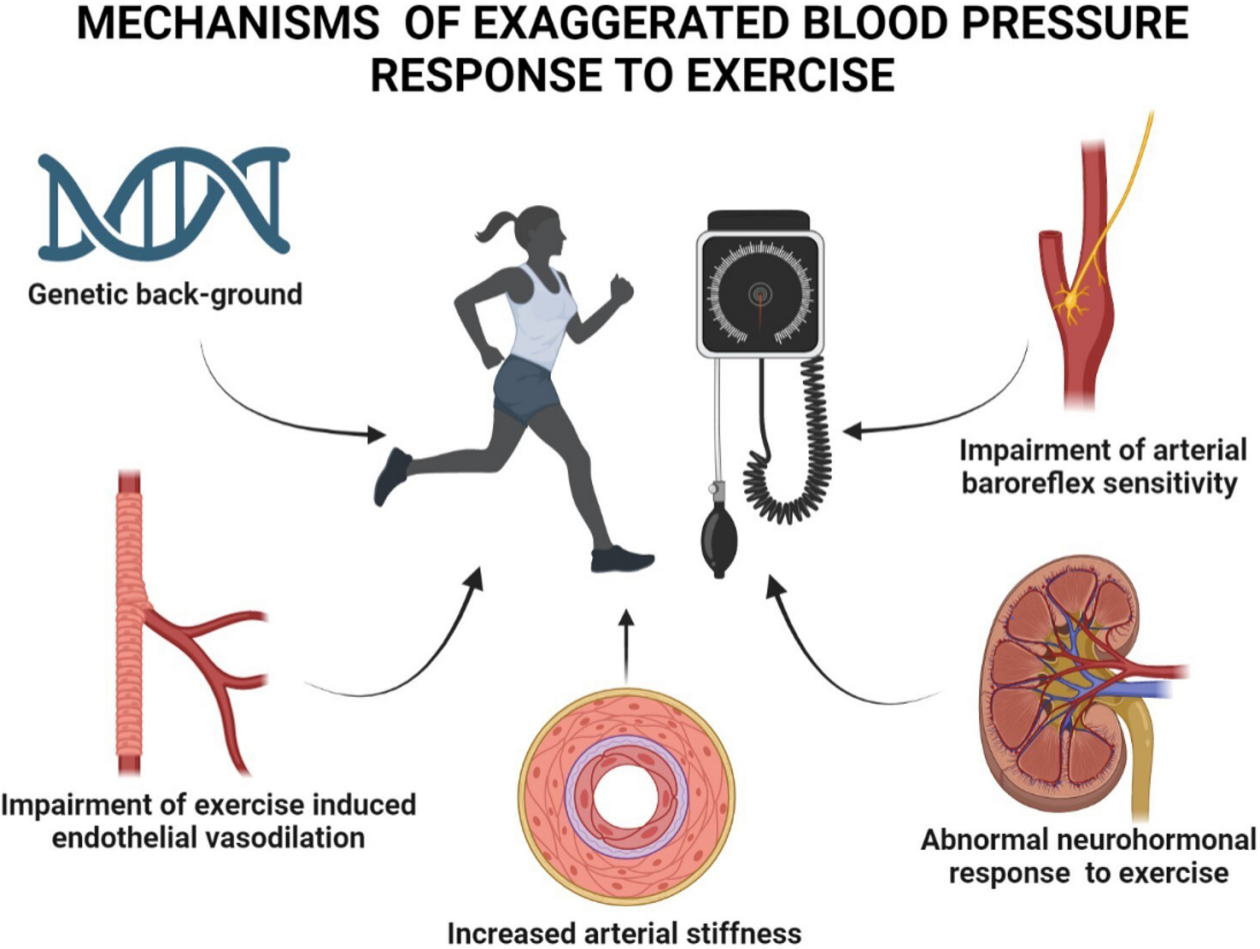
**Mechanisms and factors implicated in the development of exaggerated blood pressure response to exercise**.

Although the complex pathophysiological mechanisms of EBPR remain poorly defined, growing evidence on this issue has been collected in individuals with and without CV diseases. The multiple factors implicated in EBPR development include genetic background, endothelial function, large artery stiffness, arterial baroreflex sensitivity and neurohormonal response to exercise (Fig. 1). Emerging findings suggest that exaggerated BP and sympathetic responses to physical stimuli may reflect a deranged neural-cardiovascular control in young adults with a genetic predisposition to hypertension [28]. A greater increase in BP and muscle sympathetic nerve activity in response to static exercise has been described in young otherwise healthy adults with a family history of hypertension as compared to controls matched for age, BP and heart rate [29]. Endothelium-dependent vasodilation in large arteries represents a key adaptive response to systolic wall shear stress occurring during physical exercise [30]. Thus, endothelial dysfunction by impairing the physiological vasodilation that counterbalances the increased shear stress, may trigger EBPR. Numerous studies during the last two decades have documented an association of EBPR with endothelial dysfunction, aortic stiffness and enhanced angiotensin II rise at exercise peak [31, 32]. Stewart *et al*. [33] measured endothelial vasodilator function, assessed as brachial artery flow-mediated vasodilation (FMD), in untreated individuals with high normal BP or mild hypertension. The authors documented that FMD was the only independent correlate of the difference between resting and maximal pulse pressure in both sexes.

Increased arterial stiffness resulting in a reduced buffer function of large arteries may, in turn, enhance excessive BP increments during exercise. A recent study carried out in 92 untreated normotensive men without a history of CV disease revealed that, compared with individuals with a normal response, those with an EBPR exhibited a significantly higher brachial-ankle pulse wave velocity (1412 ± 158 vs 1250 ± 140 cm/s), after adjusting for several confounders [34]. Activation of the renin-angiotensin-aldosterone system (RAAS) and sympathetic nervous system during exercise and their involvement in EBPR has been reported by different research groups [35, 36]. In particular, greater increases in angiotensin II levels during exercise have been reported in individuals with EBPR when compared to age and gender matched controls with normal BP response [37]. The parallel increase in norepinephrine and epinephrine observed in the study by Shim *et al*. [37] supports the view that both RAAS and sympathoadrenal system mutually interact in determining BP changes during physical exercise. The increased activity of both systems leading to peripheral arterial vasoconstriction may contribute to EBPR during exercise [38]. In this regard, studies conducted in hypertensive patients, in individuals with high-normal BP as well as in elderly men have suggested that hyper-activation of muscle chemoreceptors may attenuate functional sympatholysis, enhancing the vasoconstriction, which, in combination with increased cardiac output, is responsible of EBPR [39, 40]. Finally, it should be emphasized that the mechanisms of EBPR vary in relation to the intensity of exercise. The largest part of evidence about the relationship between EBPR and outcomes is based on observations made at moderate workloads. BP recorded during submaximal exercise is likely indicative of BP variantions during daily life as reflects the usual dynamic activities [25]. Of note, BP at peak of maximal exercise may represent a confounding factor for prognostic predictions, as these protocols usually select individuals with elevated cardiopulmonary fitness and, therefore, at lower CV risk [41].

## 4. Clinical Correlates

Main research lines addressing the factors associated with EBPR have investigated the relationship between masked hypertension and BP changes during exercise. Masked hypertension is a condition characterized by normal BP values measured in the medical environment but elevated home or ambulatory BP monitoring (ABPM) values [42]. This term was coined in the early 2000’s by Pickering *et al*. [43] in order to define a hypertensive status not identified by routine office BP measurements. Since then, a large body of evidence has accumulated on the adverse clinical and prognostic significance of masked hypertension. Numerous cross-sectional and longitudinal investigations demonstrated that, compared to normotensive individuals, those with masked hypertension have an increased risk of hypertension-mediated organ damage, CV morbidity and mortality substantially overlapping that of sustained hypertensive patients [44, 45, 46]. The pathophysiological mechanisms underlying these observations are related to the fact that out-of-office BP, either monitored at home or in dynamic conditions over 24 h, has a closer relationship with CV events and a greater predictive value for adverse outcomes compared to office BP [47]. One of the first studies to investigate the relationship between masked hypertension and EBPR was published a decade ago by Sharman *et al*. [48]. Among 72 non-diabetic individuals free of CV disease with EBPR to maximal treadmill exercise (i.e., SBP ≥210 mmHg in men and ≥190 mmHg in women and/or DBP ≥105 mmHg in both sexes), the authors found that out-of-office hypertension, defined as daytime BP values ≥135/85 mmHg during 24 h ABPM, was present in the majority of the study population (58%). The prevalence of masked hypertension detected in studies conducted with similar protocols in both non-diabetic and diabetic subjects varied between 28 and 41% of the population study, thus indicating that excessive BP elevation induced by exercise occurs more frequently in individuals with hypertension unrecognized by office BP measurements [49, 50].

The presence of subclinical target organ damage in individuals with normal office BP at rest has been proven to be associated with EBPR; in particular, left ventricular hypertrophy (LVH) or concentric remodelling have been related to this abnormal BP phenotype. In a pioneering study performed in 1978 including participants to Framingham Heart Study free of CV disease and not taking any antihypertensive or CV medication, individuals with an EBPR to exercise exhibited a 10% higher LV mass than those with normal SBP responses to exercise [51]. In the previously mentioned study by Sharman *et al*. [48] patients with EBPR had significantly higher values of LV mass index (41.5 ± 8.7 vs 35.9 ± 8.5 g/$m^{2.7}$) and relative wall thickness (0.42 ± 0.09 vs 0.37 ± 0.06) than their counterparts with normal exercise BP. The association between EBPR and LVH has also been reported in the competitive sport setting. In a large cohort of 1137 athletes (mean age 21 years; 35% females) without known arterial hypertension, LVH was approximately two-fold more frequent in athletes with EBPR, defined by ACSM guidelines, than in counterparts without EBPR [27].

## 5. Prognostic Significance

Two lines of research, based on prospective studies, investigated the predictive significance of EPBR targeting its association with: (I) new-onset hypertension and (II) fatal and non-fatal CV events.

The Coronary Artery Risk Development in Young Adults (CARDIA) study was the first investigation to address the association between EBPR and incident hypertension in 3741 normotensive individuals undergoing treadmill testing [52]. EBPR was found in 687 participants (18%) who exhibited 5 mmHg higher SBP and 1 mmHg higher DBP; after a 5-year follow-up period (*p *< 0.005); these subjects were 1.70 times more likely to develop hypertension than those with normal BP response (*p *< 0.001). Although the increase in SBP associated with EBPR, after adjusting for major confounders, was small (1 to 3 mmHg), these sustained increments over time may result in a significant increased incidence of hypertension. In the late 1990s, the Framingham Heart Study provided a new piece of information on the relation of EBPR during graded treadmill test with the risk of future hypertension [53]. Over 8 years of follow-up new onset hypertension occurred in 28% of men and 16% of women. Interestingly, the most important exercise predictor of new-onset hypertension in both sexes was DBP recorded at peak exercise rather than SBP. Furthermore, a delayed recovery SBP response also predicted incident hypertension in men. The prognostic significance of EBPR to exercise has recently been addressed in different ethnic and clinical settings. In a study performed in a Japanese cohort totalling 733 male middle-aged individuals, SBP measured at the end of a 10-year follow-up period was more closely related to exercise SBP (β = 0.271, *p *< 0.001) than to resting SBP (β = 0.148, *p *< 0.001) [54]. Office BP after 10 years was 123 ± 12/79 ± 7 mmHg in individuals with low SBP response to exercise (<180 mmHg), 127 ± 13/81 ± 8 mmHg in those with moderate response (180–199 mmHg), and 134 ± 15/84 ± 10 mmHg in the group with high response (≥200 mmHg).

The value of EBPR in predicting future hypertension has also been demonstrated in the setting of athletes which, by definition, includes healthy subjects trained to perform intense physical activity. One hundred and forty-one normotensive athletes with EBPR to exercise were compared to 141 normotensive athletes with normal BP response matched for gender, age, body size, and type of sport [55]. A total of 19 athletes belonging to EBPR group developed hypertension (13.5%) compared with 5 of the normal BP response group (6.5%) during a mean follow-up period of 6.5 years.

Finally, moving from single studies to meta-analysis, the findings of a systematic review by Keller *et al*. [56] based on 18 prospective and retrospective studies including 35,151 healthy normotensive participants undergoing cardiopulmonary testing revealed a significant association between EBPR to exercise (systolic, diastolic or both) and new onset hypertension over a follow-up period lasting between 2 and 14 years regardless of the wide heterogeneity of the criteria used to define the EBPR.

The notion that EBPR in otherwise normotensive individuals may represent a risk factor for cardiovascular disease has been known for over thirty years. The Paris Prospective Study was among the first to investigate this topic by analysing the data collected in 4907 normotensive healthy middle-aged men over a mean followed-up period of 17 years [15]. The magnitude of exercise-induced SBP elevation was significantly associated with incident CV events and all-cause death, regardless of several major confounders including LVH. The value of SBP recorded during the maximal stress test for prediction of all-causes mortality, CV disease, and coronary heart disease was assessed in a large population-based sample of 20,387 men and 6234 women living in Dallas during a 8-year follow-up period [57]. In men, the adjusted risks of all-cause mortality for quartiles 3 and 4 of maximal SBP, compared to the lowest quartile, were: 1.36 (1.01–1.85), and 1.37 (0.98–1.92), respectively. This was also the case for the risk of CV disease and coronary morbidity and mortality. Similar findings for all-cause and CV mortality were observed in women across maximal SBP quartiles. Recently, the Oslo Ischemia Study provided further evidence of a positive association between the magnitude of BP responses to moderate exercise and the risk of CV disease and mortality [58].

Unlike the above mentioned studies, a recent report by Zafrir *et al*. [59], based on a retrospective analysis of 14,792 individuals followed for over 6 years, showed that the excessive increase in SBP during exercise did not predict CV disease and mortality after adjustment for confounding factors. Of note, when the SBP quartiles measured during the stress test were taken into account, the analysis documented that individuals belonging to the highest quartile were at higher risk than those in the lowest quartile.

A couple of meta-analyses have confirmed the unfavourable prognostic significance of EBPR. Pooled data from 12 studies including 46,314 individuals without overt cardiac disease suggested that the hypertensive response to an exercise of moderate intensity carried a 36% higher rate of CV events and mortality (95% CI 1.02–1.83, *p* = 0.04) compared to normal BP response [17]. The meta-analysis by Perçuku *et al*. [25], carried out in 47,188 normotensive individuals from 8 studies, showed that individuals with EBPR to exercise had a greater risk of CV death and coronary disease (HR: 1.36, *p *< 0.001) [25]. Finally, the predictive value of EBPR with respect to specific outcomes, such as coronary artery disease, sudden cardiac death and stroke, deserves to be briefly commented. The participants to the Gothemburg Study with EBPR exhibited a significant increased risk of stroke but not of myocardial infarction [60]. On the contrary, an association between EBPR and incident acute coronary syndrome and myocardial infarction was reported in the meta-analysis by Perçuku *et al*. [25]. Among the Framingham Offspring Study participants (n = 2066, mean age 58 years, 53% women) higher DBP and lower pulse pressure values during submaximal treadmill test were associated with an increased risk of heart failure over a median follow-up of 16.8 years [61]. Laukkanen *et al*. [62] failed to find an independent relationship between SBP during recovery from a symptom-limited exercise and sudden cardiac death after adjiusting for resting SBP and other CV risk factors.

## 6. Clinical Management

EBPR documented during exercise in normotensive subjects without history of hypertension and in treated hypertensives may require further diagnostic investigations, bearing in mind, however, that this BP phenotype is not a fully reproducible clinical trait. Grossman *et al*. [63], evaluating the data of exercise tests performed during annual health examinations for five consecutive years in 69 normotensive patients with high normal BP levels, found that only 11 patients (21.5%) out of the whole baseline EBPR group exhibited the same abnormal BP response during subsequent tests. Although limited data on EBPR reproducibility over time do not allow to correctly estimate the clinical relevance of this BP phenotype, the significance of EBPR in single patients should be evaluated in order to exclude both masked hypertension or uncontrolled masked hypertension. A comprehensive diagnostic approach in these patients should include BP measurements out-side the office environment (preferentially in dynamic conditions with the ABPM) and the search for subclinical hypertension mediated organ damage [64]. Available evidence of an association between EBPR, elevated ABPM values and/or target organ damage such as concentric remodelling, LVH, increased arterial stiffness, and carotid atherosclerosis should direct the clinician to plan a therapeutic intervention based on lifestyle modifications and drug treatment [65, 66, 67]. A large body of evidence supports the view that regular physical activity may favourably affect endothelial function, sympathetic activity, arterial stiffness and consequently BP levels both in normotensive individuals and hypertensive patients. Lifestyle modification programs consisting of aerobic exercise and diet counselling have been shown to reduce exercise-induced SBP elevation, improve arterial stiffness and nitric oxide bioavailability even in short-term studies (i.e., 12 weeks) [68]. Of note, the reversibility of EBPR to exercise was found to be less evident in elderly patients [69]. Nonetheless, non-pharmacological treatment of hemodynamic changes associated with EBPR represents the first mandatory step in sedentary overweight/obese individuals without history of hypertension. As for antihypertensive treatment, this option should be considered when EBPR is associated with masked hypertension, according to guideline recommendations. In treated patients with normal office BP at rest, but elevated BP during exercise, optimal treatment strategies are still uncertain [70]. As RAAS activation and increased adrenergic tone may affect vascular and myocardial responses to exercise, angiotensin converting enzyme inhibitors, angiotensin receptor antagonists and beta-blockers are the drugs of choice for hypertensive patients with EBPR [71]. Furthermore, it is useful to underline that in diabetic patients the improvement of metabolic profile may reduce and even normalize EBPR [72]. In addition, the insulin sensitizer rosiglitazone has been reported to play a beneficial effect on resting BP as well as on BP response to exercise in men with type 2 diabetes mellitus and coronary artery disease, especially in those with EBPR [73]. Finally, weight loss after bariatric surgery, a treatment increasingly used in morbid obesity, has been shown to effectively reduce the high prevalence of EBPR in these patients [74]. It is worth mentioning, however, that evidence on the effects of non-pharmacological and pharmacological interventions on CV outcomes in patients with EBPR is still lacking.

## 7. Conclusions

EBPR detected during ECG stress test is a common condition both in normotensive individuals without a known history of hypertension and in treated hypertensives with normal resting BP. Growing evidence suggests that EBPR is related to several CV risk factors such as endothelial and large artery dysfunction, increased sympathetic tone, metabolic alterations and obesity [75]. In clinical perspectives, EBPR may be regarded as a marker of multiple conditions, including masked hypertension, poor BP control, subclinical target organ damage and adverse CV outcomes [76, 77]. When this condition is ignored, CV risk is underestimated in a large proportion of the population undergoing stress testing, thus contributing to the increased burden of CV diseases with their public health consequences. It should be underlined, however, that some methodological and clinical aspects of this condition remain poorly defined, in particular clinical indications of stress test, diagnostic criteria of EBPR and screening criteria of individuals to unmask this condition. Furthermore, the role of EBPR treatment as an effective therapeutic target for reducing CV risk remains to be clarified. Thus, further studies are needed to investigate this important issue in order to prevent CV complications associated with EBPR.
